# Enhancing X‑ray Sensitivity via the Antenna Effect in Quantum Shells with Multiexciton Emission

**DOI:** 10.1021/acsnano.5c21745

**Published:** 2026-01-20

**Authors:** Jian-Xin Wang, Issatay Nadinov, Amelia Waters, Simil Thomas, Xin Zhu, Renqian Zhou, Wentao Wu, Tengyue He, Osman M. Bakr, Husam N. Alshareef, Mikhail Zamkov, Anton V. Malko, Omar F. Mohammed

**Affiliations:** † Center for Renewable Energy and Storage Technologies (CREST), Division of Physical Sciences and Engineering, 127355 King Abdullah University of Science and Technology (KAUST), Thuwal 23955-6900, Kingdom of Saudi Arabia; ‡ Department of Physics, 12335 The University of Texas at Dallas, Richardson, Texas 75080, United States; § The Center for Photochemical Sciences and Department of Physics, 1888 Bowling Green State University, Bowling Green, Ohio 43403, United States

**Keywords:** quantum shells, multiexciton emission, X-ray imaging, antenna effect, energy transfer

## Abstract

Quantum shells (QSs) with efficient multiexciton emission can generate multiple excitons per particle under high-energy excitation, thereby improving exciton utilization under intense X-ray exposure and offering strong potential for X-ray-based scintillation applications. However, these QSs are typically composed of low-atomic-number (*Z*) elements, which substantially limits their X-ray absorption efficiency and leads to poor X-ray sensitivity. Here, we overcome this fundamental limitation by introducing a high-*Z* antenna-sensitization strategy that couples QSs to heavy-element molecular absorbers, which act as X-ray harvesting centers and funnel energy into the QSs via efficient interfacial transfer. By combining enhanced X-ray absorption with efficient interfacial energy transfer and improved exciton utilization, we achieve more than an order-of-magnitude increase in multiexciton-driven QS radioluminescence (RL) relative to pristine shells. Additionally, a high X-ray imaging resolution of 25.2 lp mm^–1^ was achieved, exceeding the performance of most previously reported X-ray imaging scintillators. These findings offer a promising design strategy for advancing QS-based materials toward high-performance X-ray imaging applications.

## Graphical abstract

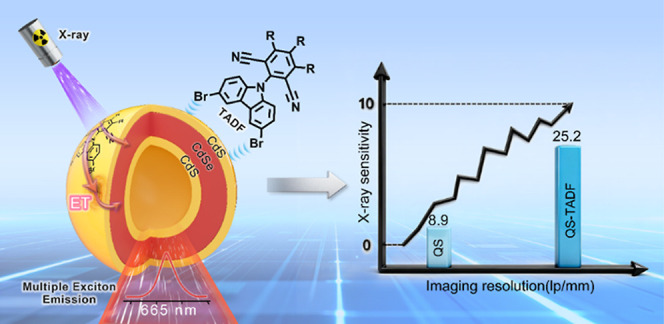

## Introduction

X-ray imaging technology is vital for applications in medical diagnostics and security screening, where it directly supports human health and safety, and in high-energy physics, where it enables fundamental investigations of matter.^1–7^ Its effectiveness in these applications critically relies on scintillator materials capable of efficiently converting high-energy X-ray photons into visible light, enabling the visualization of internal structures in both biological organisms and complex devices.^8–15^ Traditionally, the most popular scintillators have been based on inorganic crystals and perovskites; however, these materials often require harsh synthesis conditions, lack long-term stability, and present challenges for large-area fabrication.^16–20^ In response, organic materials have emerged as promising alternatives owing to their tunable photophysical properties, high stability, and compatibility with flexible fabrication processes. Despite these advantages, their poor exciton utilization limits X-ray sensitivity and diminishes their practical viability.^21–26^ Consequently, developing novel scintillators integrating high efficiency, fast response, enhanced energy and spatial resolution, long-term stability, and scalability remains a critical objective.

Among the explored scintillator candidates, quantum shells (QSs)—a new type of nanocrystals that offers enhanced multiexciton (MX) emission, has emerged as a promising solution for improving photoconversion efficiency, especially under X-ray excitation, where exciton utilization is markedly improved.^27–29^ The overall performance of QS scintillators is further enhanced by their emission tunability in the deep-red spectral range. Their large Stokes shifts reduce self-absorption, further enhancing photon utilization. In addition to their favorable emission characteristics, these nanocrystals are compatible with solution processing and scalable fabrication, opening up new avenues for the design and development of next-generation scintillators.^30–33^ However, their X-ray absorption remains limited because most QSs are synthesized from low-atomic-number elements (e.g., Cd, S, Se), which in turn reduces their radioluminescence (RL) efficiency. To overcome this drawback, incorporating antenna molecules with strong X-ray absorption, along with efficient capability for light harvesting and energy transfer to the QSs, offers a promising strategy.^34–39^

This study presents the first QS scintillator based on multiple exciton emission, incorporating small molecules with strong X-ray absorption as antenna units to enable efficient RL (Figure 1). Spectroscopic analysis, including steady-state and ultrafast transient measurements, confirms that the QS retains its MX emission after integration, which contributes notably to its enhanced RL performance. Leveraging this, the system achieves efficient X-ray absorption, improved exciton utilization, and a 10-fold enhancement in RL output compared to the QS itself. The system also delivers an X-ray imaging resolution of 25.2 lp mm^–1^, surpassing most previously reported X-ray imaging scintillators. These findings lay the foundation for a viable strategy for designing high-efficiency QS-based X-ray energy conversion systems.

**Figure 1:**
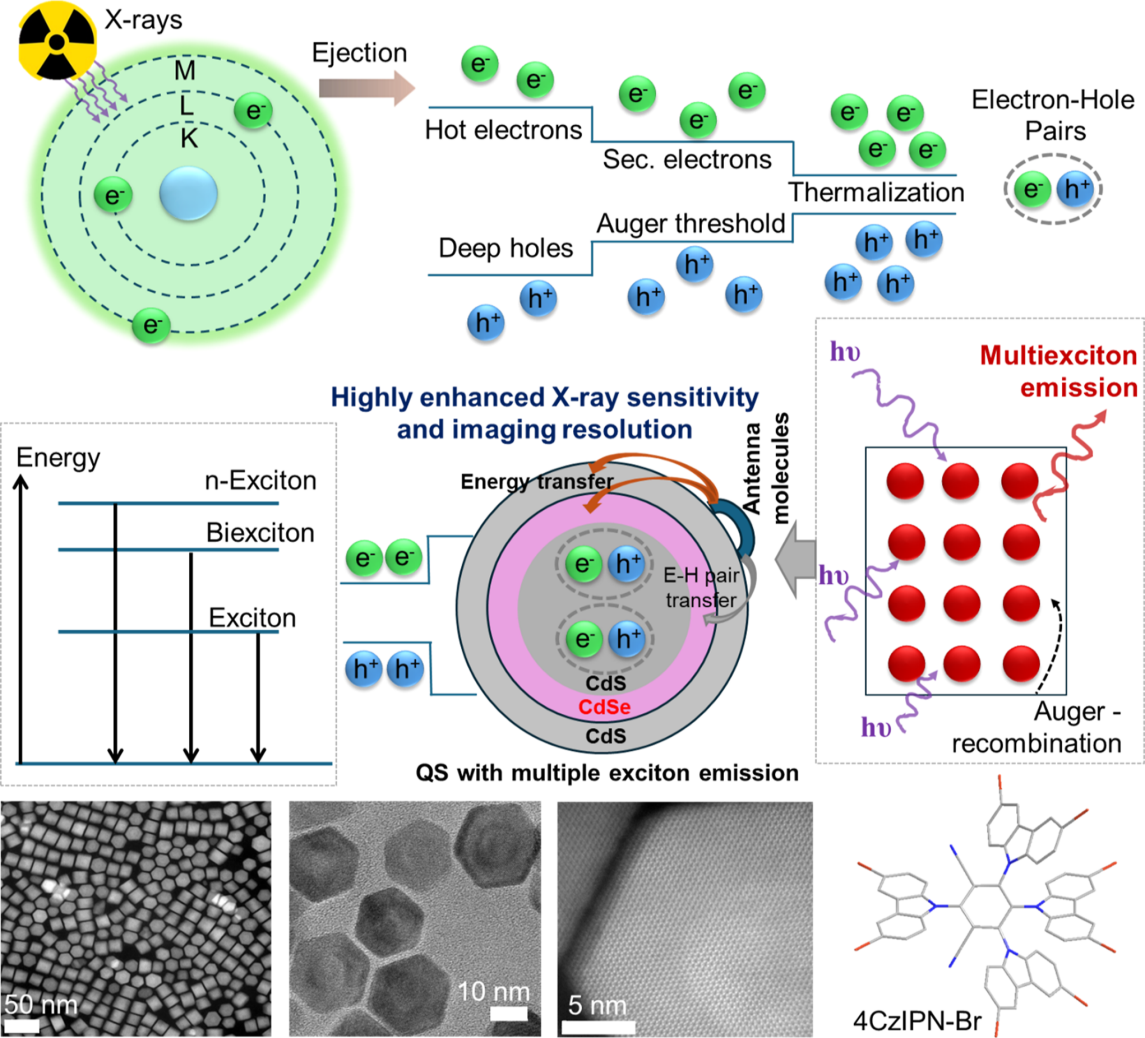
Schematic of the QS-based X-ray scintillation process, highlighting MX emission and an efficient energy transfer pathway, along with a transmission electron microscopy (TEM) image of the QSs and the molecular structure of 4CzIPN-Br.

## Results and Discussion

Because multiple exciton emission in QSs is efficiently triggered by high-energy excitation, X-ray photon absorption by the QSs (Figures 1 and S1) is expected to generate multiple electron–hole pairs (i.e., MXs). These MXs may subsequently decay radiatively, contributing to RL, or nonradiatively via Auger recombination. This latter pathway poses a major limitation in strongly confined colloidal nanocrystals owing to ultrafast recombination lifetimes (hundreds of picoseconds), which outcompete radiative emission. To investigate the excited-state dynamics and elucidate the species and recombination pathways of MX states, we conducted excitation-fluence-dependent, time-resolved femtosecond transient absorption (fs-TA) measurements on dilute QS solutions. This approach enabled the monitoring of excited-state population dynamics and differentiation between single and MX contributions across varying excitation intensities.

In these experiments, the change in absorption (Δα = α – α_0_) induced by an fs laser pulse was monitored using a broadband white-light pulse continuum. Figure 2a presents a series of power-dependent TA spectra for 6 nm core CdS–CdSe–CdS QSs, expressed in terms of both fluence and the average number of electron–hole pairs, ⟨*N*_eh_⟩ = *fs*. Here, *f* denotes the excitation fluence in photons/cm^2^, and *s* = 3.5 × 10^–13^ cm^2^ represents the absorption cross-section of a single particle, estimated from TEM size data using standard analysis.^40^ The TA spectra exhibit two distinct bleach (negative absorption) features centered at 630 and 525 nm, corresponding to absorption within the CdSe shell (quantum-confined inner layer) and CdS regions (core and outer capping shell), respectively. Both features appear simultaneously owing to concurrent photon absorption by different shell regions but decay on distinct time scales, as illustrated in the mapping image shown in Figure 2b. Acquired at a much higher excitation fluence (100 μJ/cm^2^), this mapping reveals that charge carriers in the CdS domain decay more rapidly, primarily owing to Auger recombination and carrier relaxation into the energetically lower-lying CdSe shell.

**Figure 2:**
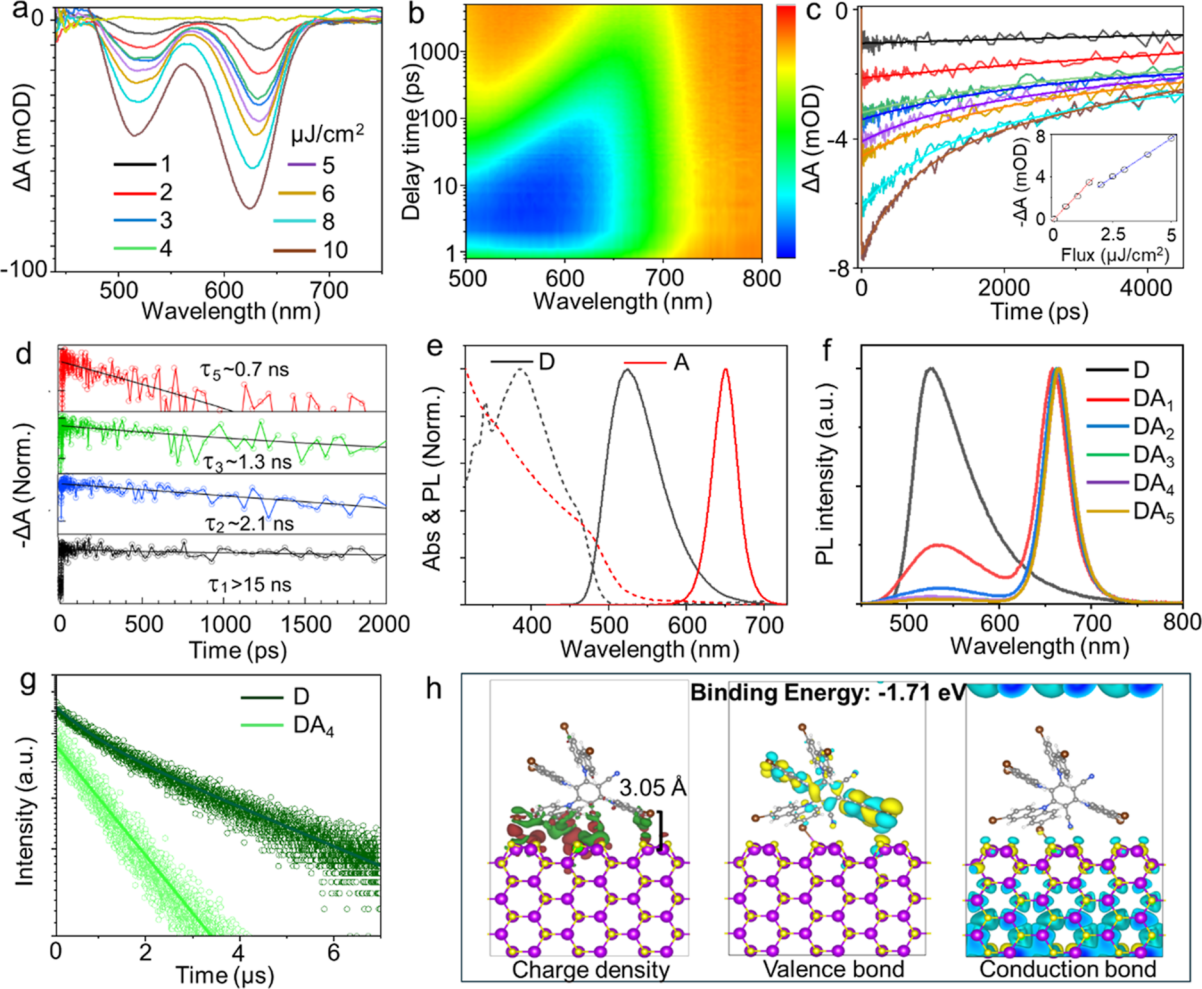
(a) Power-dependent TA spectra of the QS (1 μJ/cm^2^) corresponding to ⟨Neh⟩ = 0.7 electron−hole pairs. (b) Corresponding mapping acquired at 400 nm with an incident fluence of 100 μJ/cm^2^. (c) Population-dependent TA dynamics recorded at different photon fluences. The inset displays the relationship between excitation fluence and signal intensity. (d) Exciton and multiexciton decay kinetics. (e) Absorption and photoluminescence (PL) spectra of the QS (acceptor, A) and 4CzIPN-Br (donor, D). (f) PL spectra and (g) PL decay profile measured at 525 nm. (h) Charge-density difference plots and wave function of the valence and conduction band edges at the Γ-point for the QS–4CzIPN-Br complex obtained from DFT calculations.

To characterize Auger recombination dynamics, we extracted the carrier decay profiles at the lowest-energy bleach feature, as illustrated in Figure 2c. At the lowest pump fluence of ⟨*N*_eh_⟩ = 0.7 electron–hole pairs (corresponding to 1 μJ/cm^2^), the decay profile exhibited a long, nearly monoexponential trend with a characteristic decay time of τ_*x*_ > 15 ns, although a more precise determination was limited by the accessible delay-time window. This behavior is consistent with a single exciton (X) transition associated with the low-energy bleach feature possessing a long radiative lifetime, as measured by PL decay (Figure S2). With increasing excitation power, faster decay components progressively emerged, corresponding to the formation of excitonic complexes such as biexcitons and higher-order MXs. To extract Auger lifetimes, we recorded exciton population dynamics across carrier densities of ⟨*N*_eh_⟩ = 1.5–5.6 electron–hole pairs and applied a simple subtractive procedure to approximate the monoexponential decays for each consecutive MX species.^28,41^ As displayed in Figure 2d, although the extracted Auger lifetimes for 2, 3, and 5 electron–hole pairs became progressively shorter, they remained on the order of several nanoseconds, indicating that Auger recombination was strongly suppressed.^28^ Consequently, the excitation of multiple exciton pairs by a high-energy X-ray pulse is expected to contribute to efficient RL and enhanced scintillation performance.

However, the lack of heavy atoms in QSs results in a relatively low X-ray absorption cross-section, which restricts both their X-ray sensitivity and imaging resolution. While the direct incorporation of heavy elements into QSs could improve absorption, this approach presents considerable challenges: it often disrupts the crystalline structure and introduces nonradiative decay channels through the heavy atom effect, leading to pronounced quenching of emission efficiency. To circumvent these limitations, we introduce an energy transfer strategy that employs antenna molecules (4CzIPN-Br) containing high-atomic-number elements. These molecules can be grafted onto the QS surface (Figure S1), where they serve as effective X-ray absorbers and nonradiatively transfer energy to the QS, thereby enhancing scintillation efficiency (Figure 1). This strategy enables the synergistic integration of enhanced X-ray absorption, efficient light harvesting, and improved exciton utilization while preserving the QS’s intrinsic capacity for multiple exciton emission (Figures S3–S6). Collectively, these attributes lead to a substantial improvement in both X-ray sensitivity and imaging resolution.

As illustrated in Figure 2e, the antenna molecules, possess a broad absorption band below 500 nm and an emission band spanning 500–600 nm that overlaps with the absorption spectrum of the QS nanoparticles. The absorption spectrum of CdS/CdSe/CdS nanoparticles comprises two regions: a low-energy, low-intensity band between 530 and 650 nm arising from the CdSe QS and a high-energy, high-intensity band between 350 and 520 nm associated with absorption in the CdS core and outer layer. High-resolution TEM images and prior data for the medium-core QS sample^27,28^ indicate a core radius of *R*_c_ = 3 nm, a CdSe shell thickness of *H*_Se_ = 2 nm, and an outer CdS layer thickness of *H*_S_ = 4.6 nm. Accordingly, the outer CdS layer accounts for approximately 86% of the total volume, whereas the CdSe QS comprises approximately 11%. Therefore, the high-energy absorption component of the CdS/CdSe/CdS nanoparticle primarily arises from the outer CdS layer. This layer also lies closest to the antenna molecules; consequently, most nonradiative energy transfer is directed to the CdS outer layer. Under X-ray excitation, this CdS layer accumulates multiple exciton pairs originating from both direct absorption and energy transfer. These excitons subsequently relax into the active CdSe quantum shell layer, where they contribute to MX generation for the RL signal.

To investigate the energy transfer process, we prepared donor–acceptor (D–A_*n*_) composite films, in which *n* denotes the weight percentage of acceptor QS nanoparticles. Donor antenna molecules were incorporated into a polysulfone matrix at a fixed concentration of 5 wt %. With the donor concentration held constant, the acceptor QS content was systematically increased. As depicted in Figure 2f, the donor PL intensity, normalized per acceptor, progressively decreased with increasing QS loading, indicating efficient energy transfer from the donor to the acceptor. Based on donor PL quenching, the energy transfer efficiency was estimated to reach as high as 97%. Concurrently, the PL lifetime of the donor emission, decreased from 2.18 to 0.59 μs with increasing acceptor content (Figure 2g), further confirming the energy transfer. Furthermore, DFT calculations (Figures 2h and S7) revealed a halogen-bond-like interaction between the antenna molecules and the QS, characterized by a short intermolecular distance. This proximity underpins the strong interfacial interaction and facilitates efficient energy transfer. Furthermore, an analysis of the conduction and valence band distributions revealed that the conduction band is primarily localized on the QS, while the valence band resides on the antenna molecules.

The RL spectra of the D–A_*n*_ QS composite films under X-ray excitation exhibit trends consistent with their corresponding PL spectra under UV excitation. As seen in Figure 3a, donor RL emission intensity is almost completely quenched, while acceptor RL emission is increased by ∼10 times for films with highest acceptor concentrations. This concurrent behavior further proves energy transfer to be the primary mechanism responsible for the enhanced RL from the QS composite films. To quantify the scintillation performance, the integrated RL spectra of the (D–A_4_) QS composite and pure acceptor A_4_ films were analyzed (Figure 3b) and compared to those of the standard scintillator bismuth germanium oxide (BGO). Based on this comparison, the D–A_4_ QS composite film exhibits a high light yield (LY) of approximately 21,000 photons MeV^–1^, 10 times greater than that of the pure QS film under identical experimental conditions (Figure 3c and Table S1) and twice as much as standard BGO. Another important metric for a scintillator is fast operational speed. Conventional standards have RL decay constants on the order of a microsecond, along with the rise times of a few nanoseconds.^42^ Additionally, common standards exhibit millisecond afterglow which is detrimental to fast imaging applications. Contrary, QS composite films exhibit fast RL dynamics, with proper decay times (*t*_p_) determined by MX QS decays of a few ns and afterglow times (determined when RL intensity drops to <1% of the initial value) on tens of ns as seen in Figure S8.

**Figure 3:**
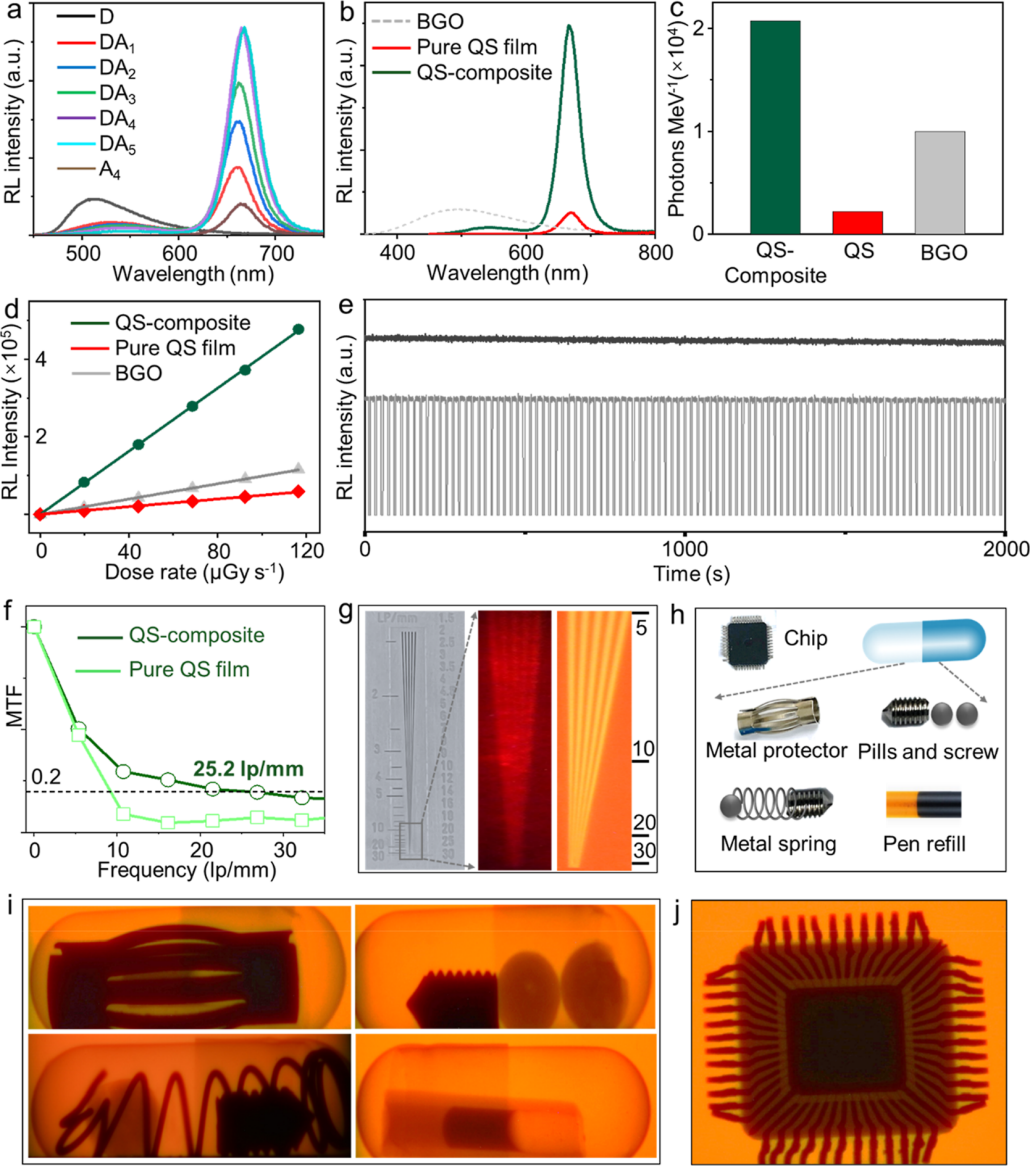
(a) RL spectra of the QS composite system (D–A_*n*_), where *n* denotes the weight percentage of A, and D was incorporated into a polysulfone matrix at a fixed loading of 5 wt %. (b) RL spectral comparison between the QS composite and standard scintillator bismuth germanium oxide (BGO). (c) Corresponding comparison of light yield. (d) Dose rate–response curves showing RL intensity as a function of X-ray dose rate for BGO and the QS composite. (e) Photostability of the QS composite under continuous and cyclic on–off X-ray irradiation. (f) Modulation transfer function (MTF) of a QS-based X-ray scintillation screen. (g) Corresponding line-pair phantom image acquired under X-ray excitation. (h) Patterned elemental masks used for X-ray imaging demonstrations, and (i,j) their corresponding X-ray images.

In addition, the D–A QS composites display a linear correlation between RL intensity and X-ray dose rate across a broad range, ensuring accurate detection of varying X-ray exposure levels—an essential requirement for quantitative imaging and precise dosimetry (Figures 3d and S9). The photostability of the composites was further evaluated under continuous X-ray irradiation at a dose rate of 0.25 mGy/s for 2000 s, during which the RL intensity retained over 98% of its initial value (Figures 3e and S10). Moreover, the RL response remained stable after more than 100 cycles of repeated on–off X-ray excitations, demonstrating excellent operational durability. Moreover, based on RL measurements of the D–A QS-composite film immediately after preparation and after 3 months of storage, only a slight decrease in RL intensity was observed (Figure S10), indicating the good storage stability of the QS-composite system. These stability characteristics highlight the strong potential of the QS-composite for reliable and long-term use in X-ray imaging and continuous security screening applications.

Owing to the excellent X-ray sensitivity of the D–A QS composites, the fabricated D–A QS scintillating screen achieved an imaging resolution of 25.2 lp mm^–1^ at an MTF value of 0.2 (Figure 3f). This resolution surpasses that of most reported organic and commercial scintillators (Table S2) and was further validated by line-pair card measurements (Figure 3g). The practical utility of the scintillating screen was further confirmed through a series of capsule imaging tests (Figure 3h), demonstrating its potential for high-resolution X-ray imaging in applied settings. Under X-ray excitation, a metal object sealed within a capsule was clearly visualized on the D–A QS scintillation screen. Furthermore, various items, including a metal screw and two pills, were successfully imaged. More complex combinations, including a spring, a metal screw, and a pill enclosed within a single capsule, were clearly resolved. A ballpoint pen refill was imaged, and the ink inside the barrel was distinctly visible (Figure 3i). The internal structure of an electronic chip was clearly resolved, with its plastic casing appearing as a distinguishable region (Figure 3j). These demonstrations collectively highlight the exceptional spatial resolution and sensitivity of the D–A QS composite, underscoring its strong potential as a high-precision material for advanced X-ray imaging applications.

## Conclusions

This study demonstrates that QSs, engineered for efficient MX emission, can be transformed into high-performance scintillators for X-ray imaging by coupling them to high-*Z* molecular “antenna” absorbers. In this donor–acceptor architecture, small heavy-atom molecules act as X-ray harvesting centers and funnel their energy into QSs with minimal loss, thereby simultaneously enhancing X-ray stopping power and exciton utilization within the same composite. Leveraging the combined advantages of MX emission and energy transfer, we achieved an X-ray-to-visible light conversion efficiency of approximately 21,000 photons/MeV, corresponding to nearly a ten-fold enhancement over unmodified QSs. The D–A QS composite displayed a high X-ray imaging resolution of 25.2 lp mm^–1^, surpassing the performance of most colloidal and many commercial scintillation platforms and highlighting its suitability for advanced imaging across diverse application domains. This antenna–QS strategy is, in principle, broadly extendable to other material systems by rationally selecting antenna molecules with strong X-ray absorption cross sections and well-matched energy levels to enable efficient interfacial energy transfer to the luminescent core. Through molecular design of antennas with stronger binding affinity and surface engineering of QSs, effective coupling between the antenna and QS can be achieved. Although challenges such as interfacial stability, energy-level alignment, and long-term durability under X-ray irradiation may arise, these issues can be addressed through optimized chemical anchoring strategies and compositional tuning. Overall, this modular antenna–QS design provides a versatile and general platform for the development of high-performance X-ray imaging scintillators.

## Methods

### Materials

All reagents were used as received without additional purification: anhydrous acetone (99%, Amresco), cadmium oxide (CdO, 99.95%, MilliporeSigma), zinc acetate dihydrate (98%, Acros Organics), anhydrous ethanol (99%, BeanTown Chemical), hexane (ACS grade, Thermo Scientific), 1-octadecene (ODE, technical grade, 90%, MilliporeSigma), octane (98%, MilliporeSigma), 1-octanethiol (97%, Alfa Aesar), oleic acid (OA, technical grade, 90%, MilliporeSigma), oleylamine (OLAM, 70%, MilliporeSigma), dioctylamine (DOA, 97%, MilliporeSigma), rhodamine 101 inner salt (R101, 94%, Thermo Scientific), selenium powder (99.5%, 200 mesh, Thermo Scientific), sulfur powder (99.999%, Thermo Scientific), toluene (99.8%, MilliporeSigma), and tri-*n*-octylphosphine (TOP, 97%, Strem Chemical).

### Preparation of the Scintillation Screens

5 mg of 4CzIPN-Br were dissolved in 0.6 mL of chloroform, and *x* mg of QS were subsequently added. After sonicating for a few seconds, 95 – *x* mg of polysulfone (PSF) was introduced. The mixture was then shaken on a shaker for 2 h to ensure thorough mixing of 4CzIPN-Br, QS, and PSF. The resulting viscous solution was carefully coated onto quartz plates to form films. Notably, the films were covered with a beaker during solvent evaporation to ensure uniformity. The film thickness can be tuned by adjusting the concentration of the composite in chloroform and the volume of the viscous solution applied.

### Calculation of Energy Transfer Efficiency

The energy transfer efficiency was calculated through the following equation

$$\varepsilon =1-\frac{I_{DA}}{I_{D}}$$

where ε is the energy transfer efficiency; *I*_DA_ is the PL intensity at the donor emission wavelength in the donor–acceptor energy transfer system; and *I*_D_ is the PL intensity of the donor alone.

## Supplementary Material



## Notes

Detailed experimental procedures; steady-state and ultrafast spectroscopic analyses with corresponding figures; and comparative tables summarizing scintillator performance (PDF)
