# Supplementary material for: Enhancing X‑ray Sensitivity via the Antenna Effect in Quantum Shells with Multiexciton Emission
Source: ACS Nano. 2026 Jan 20;20(4):3878–85. doi: 10.1021/acsnano.5c21745 (PMC12875021; doi:10.1021/acsnano.5c21745)
Supplement: Supplementary file 1 [file nn5c21745_si_001.pdf]

Enhancing X-ray Sensitivity via the Antenna Effect in Quantum Shells with Multiexciton Emission

Jian-Xin Wang,[1] Issatay Nadinov,[1] Amelia Waters,[3] Simil Thomas,[1] Xin Zhu,[1] Renqian Zhou,[1] Wentao Wu,[1] Tengyue He,[1] Osman M. Bakr,[1] Husam N. Alshareef,[1] Mikhail Zamkov,[3] Anton V. Malko[2]* and Omar F. Mohammed[1]*

[1]Center for Renewable Energy and Storage Technologies (CREST), Division of Physical Sciences and Engineering, King Abdullah University of Science and Technology (KAUST), Thuwal 23955–6900, Kingdom of Saudi Arabia.

[2]Department of Physics, The University of Texas at Dallas, Richardson, Texas 75080, United States

[3]The Center for Photochemical Sciences and Department of Physics, Bowling Green State University, Bowling Green, Ohio 43403

Email: anton.malko@utdallas.edu and omar.abdelsaboor@kaust.edu.sa

Materials and methods

Photoluminescence measurements were collected on an Edinburgh FS-5 spectrometer. UV-Vis absorption studies were conducted with a PerkinElmer Lambda 950 UV/Vis spectrometer.

Synthesis of the quantum shells

Preparation of CdS Nanocrystals. Large CdS nanocrystals (12–16 nm diameter) were grown using a seed coalescence approach described previously,[1] with minor modifications. A mixture of 8 mL OLAM and 42 mg $CdCl_2$ was loaded into a 25 mL flask, degassed, and then placed under argon flow. The mixture was heated to 290 °C before injecting 540 nmol of 2–4 nm CdS seeds. Growth proceeded for 1 h at this temperature, after which the flask was cooled below 220 °C and quenched in a water bath. Crude products were purified by sequential precipitation with a toluene/ethanol (~1:2) mixture, followed by redispersion in toluene and centrifugation to remove grey precipitate. The purified NCs were then transferred into 5 mL ODE and 8 mL OA, heated under argon at 150 °C for 1 h, cooled, and reprecipitated as before. The resulting CdS NCs were suspended in hexane.

Growth of CdS-CdSe Core-Shell Nanocrystals. CdSe shells were deposited on CdS cores using cadmium oleate and TOP-Se precursors delivered by syringe pump. The cadmium oleate solution was prepared by dissolving 412 mg CdO in 8 mL OA and 5 mL ODE at 260 °C under argon until clear, followed by addition of 19 mL ODE. The Se precursor was formed by heating 141 mg Se with 3 mL TOP at 140 °C until fully dissolved, then diluting with 14 mL ODE. For shell growth, 540 or 1080 nmol of CdS cores were dispersed in 3 mL OLAM and 3 mL ODE within a 100 mL flask, degassed at 100 °C, and then heated under argon to 315 °C. At 270 °C, both precursors were introduced at 3 mL/h. Injection continued until the PL peak reached 630-680 nm, corresponding to ~90 min for large NCs and ~120 min for giant NCs. Following growth, the solution was cooled to room temperature and the product purified with toluene/ethanol precipitation. Final samples were dispersed in hexane.

Synthesis of CdS-CdSe-CdS quantum shells. For outer CdS shells, the cadmium oleate solution described above was employed together with an octanethiol precursor (0.4 mL octanethiol in 13.6 mL ODE). Core–shell NCs (540 or 1080 nmol) were dispersed in 3 mL OLAM and 3 mL ODE, degassed at 100 °C, and then heated under argon to 315 °C. Beginning at 270 °C, the two precursors were co-injected at 3 mL/h. Shell growth typically continued for 4–5 h, after which the

reaction mixture was annealed for an additional 30 min, cooled, and purified by centrifugation using a toluene/ethanol mixture. The final CdS-CdSe-CdS quantum shell product was collected in hexane.

Time-correlated single-photon counting (TCSPC)

TCSPC measurements were performed using a HALCYONE setup (Ultrafast Systems). Excitation at 400 nm was provided by a parametric optical amplifier (Newport, Spectra-Physics) pumped by an Astrella femtosecond laser (800 nm, 150 fs pulse duration, 1 kHz repetition rate; Coherent). The photoluminescence (PL) from the samples was collected and recollimated with a pair of parabolic mirrors, transmitted through a 450 nm long-pass filter (Newport), and focused into an optical fiber coupled to a monochromator and a photomultiplier tube detector. The excitation fluence at each wavelength was adjusted using a pair of variable neutral density filters (Thorlabs) to ensure that fewer than 1% of excitation events resulted in photon detection, thereby maintaining operation within the single-photon counting regime. TCSPC decay histograms were analyzed using the Levenberg–Marquardt fitting algorithm implemented in the Ultrafast Systems-Surface Xplorer software. The overall temporal resolution of the system was better than 120 ps.

Femtosecond transient absorption (fs-TA) measurements

The samples were excited with 400 nm pump pulses generated by directing a fraction of the 800 nm fundamental output (~150 fs, 1 kHz, 7 mJ/pulse; Astrella, Coherent) into a spectrally tunable optical parametric amplifier tuned to 400 nm. The probe continuum in the ultraviolet–visible and near-infrared (white light) was produced by focusing another portion of the 800 nm pulses into a 2 mm thick calcium fluoride (CaF_2) crystal. Prior to white-light generation, the 800 nm pulses were sent through a motorized delay stage, allowing the detection of transient species at variable pump–probe delays. The resulting white-light continuum was split into signal and reference beams and coupled into two optical fibers to enhance the signal-to-noise ratio. The excitation pump and probe beams were spatially overlapped on the sample, with the pump modulated at 500 Hz by a synchronized mechanical chopper that blocked every second pump pulse. The differential absorbance (ΔA) was recorded as a function of probe delay time and wavelength (λ). The instrument response function (IRF) of the transient absorption setup was determined to be 168 fs.

Data were analyzed using the fitting algorithms implemented in the Ultrafast Systems - Surface Xplorer software.

Computational methods.

Geometry optimizations of the QS and antenna molecule systems were performed using the Vienna Ab initio Simulation Package (VASP)[2] within the framework of density functional theory (DFT). The generalized gradient approximation (GGA) with the Perdew–Burke–Ernzerhof (PBE) exchange–correlation functional was employed throughout. A plane-wave kinetic energy cutoff of 400 eV was used for all calculations. Long-range dispersion interactions were accounted for using the DFT-D3 method of Grimme[3] with Becke–Johnson damping.[4] For the QS model, a $5 \times 5 \times 1$ supercell was sampled using a Γ-centered Monkhorst-Pack k-point mesh of $2 \times 2 \times 1$. All structures were fully relaxed until the residual Hellmann–Feynman forces on each atom were less than 0.02 eV $Å^{-1}$.

Radioluminescence (RL) measurement.

Steady-state RL spectra were collected using a spectrometer (Fluoromax-4) coupled with an X-ray tube (Tungsten target, Moxtex). The detection slit was set to 5 nm, and the X-ray outlet was positioned 1 cm away from the sample for all spectral measurements. The X-ray dose was controlled by adjusting the tube current and voltage. The RL decay profile was performed on a Edinburgh FS-5 spectrometer coupled with a X-ray chamber. The tube voltage and current used for the RL measurements were 30 kV and 50 μA, respectively.

Calculation of light yield.

Commercially standard scintillators, BGO, sourced from OST PHOTONICS, were used as reference materials to estimate the light yield of the samples. RL spectra were recorded with a spectrometer under consistent settings, ensuring identical sample and scintillator sizes. Light yield calculations were performed by integrating the X-ray-induced RL spectra of the samples and comparing them to the integrated spectra of the reference scintillators.[5-6] The tube voltage and current used for the RL measurements were 30 kV and 50 μA, respectively.

$LY_{sample} \approx LY_{ref} \times \frac{I_{sample}}{I_{ref}}$ where LY_{sample} is the light yield of the tested scintillator, LY_{ref} is the known light yield of the reference scintillator, and I_{sample} and I_{ref} are the integrated RL spectra of the sample and the reference, respectively.

X-ray image collection and processing.

The X-ray imaging tests were conducted using a homemade system that included an X-ray source, optical elements, and a camera (Nikon D7100), all enclosed in a lead box. The spatial resolution of X-ray imaging was evaluated using Modulation Transfer Function (MTF) measurements, calculated by the slanted-edge method. A sharp-edge image of an iron sheet was captured for this analysis. Using ImageJ software, the edge spread function (ESF) was derived from the edge image, and the line spread function (LSF) was obtained by differentiating the ESF. The MTF was then defined as the Fourier transform of the LSF. The tube voltage and current used for the X-ray imaging were 50 kV and 80 μA, respectively. The following formula summarizes the process: [7]

$$MTF(v) = F\big(LSF(x)\big) = F(\frac{dESF(x)}{dx})$$

Where ν is the spatial frequency.[8-9]

Supplemental Figures and Tables

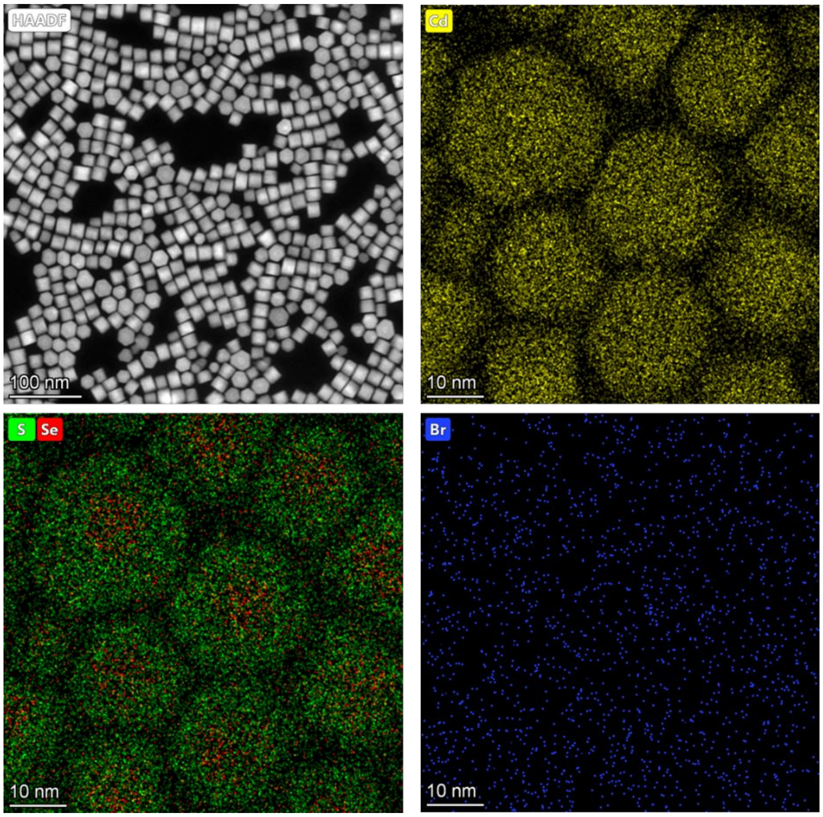

Figure S1. The STEM image and the corresponding EDS mapping of the quantum shells mixed with the antenna molecules.

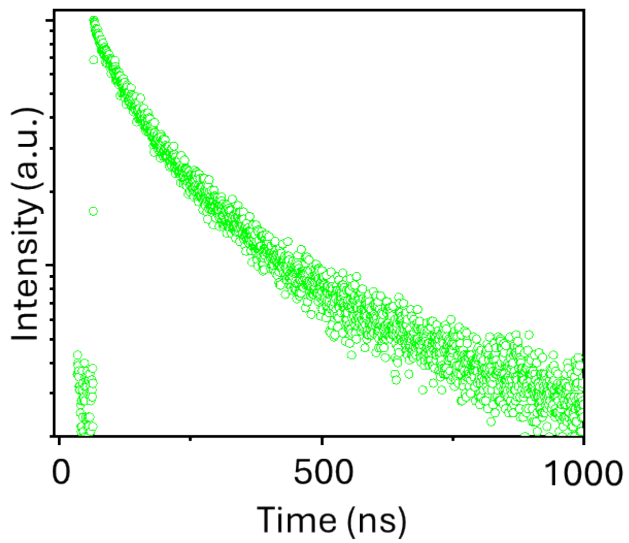

Figure S2. Photoluminescence decay profile of the QS.

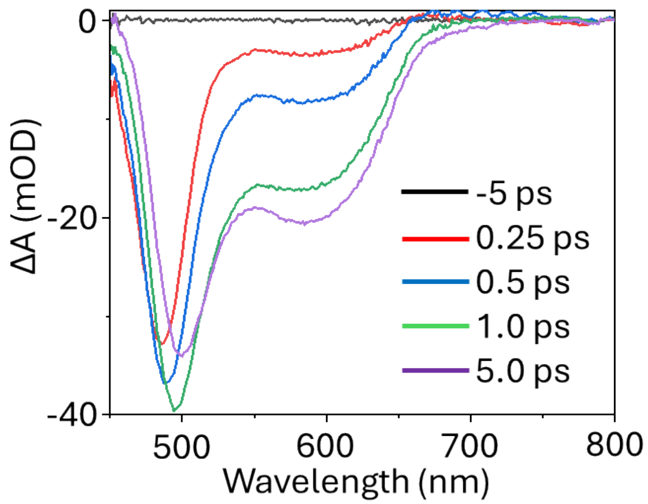

Figure S3. The transient absorption spectra of the quantum shell under 400 nm excitation with an excitation power of 100 μJ/cm^2.

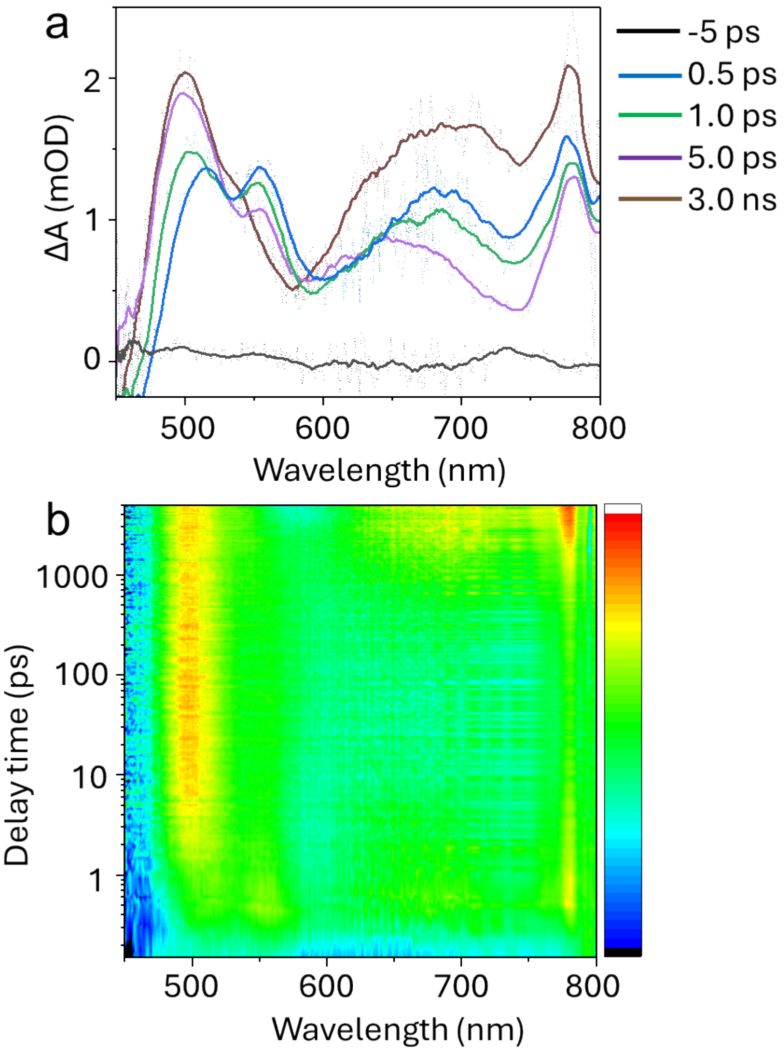

Figure S4. (a) Transient absorption spectra of the antenna molecule 4CzIPN-Br, and (b) the corresponding spectral mapping under 400 nm excitation at a power of 100 μJ/cm^2.

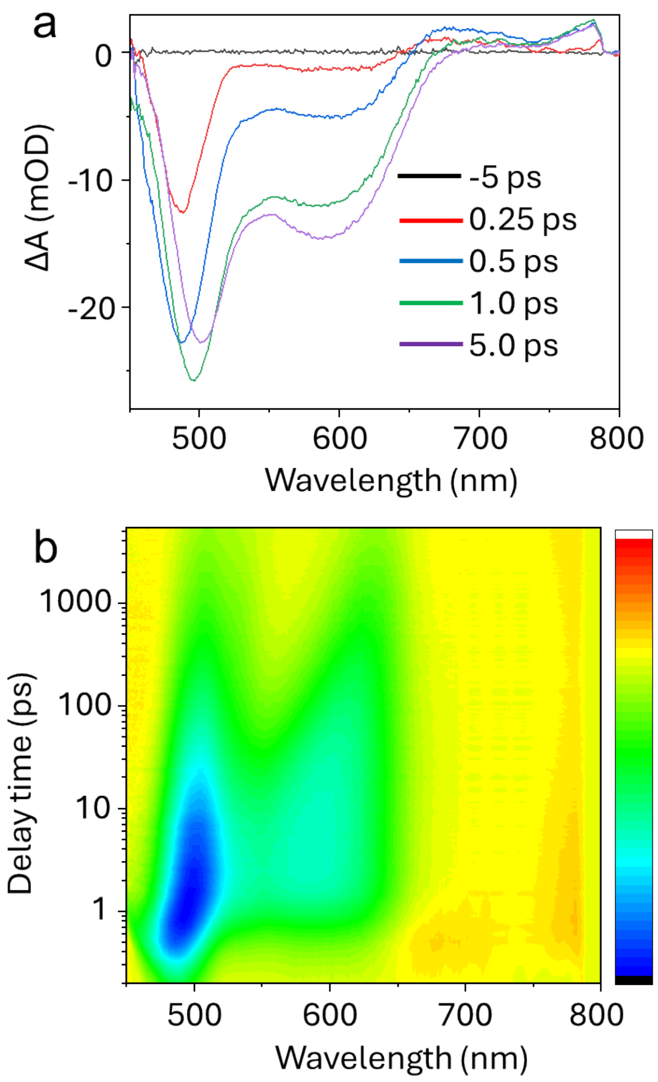

Figure S5. (a) Transient absorption spectra of the D-A QS composite, and (b) the corresponding spectral mapping under 400 nm excitation at a power of 100 μJ/cm^2.

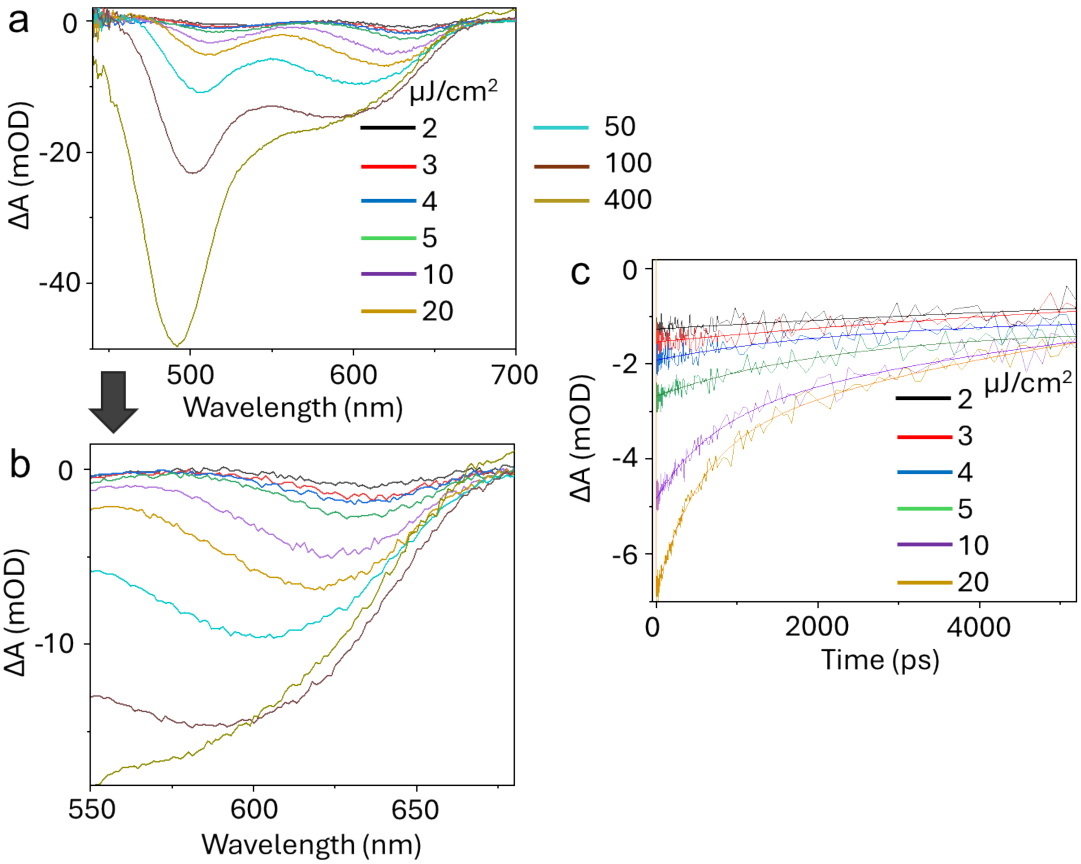

Figure S6. (a-b) Fluence-dependent transient absorption (TA) spectra of the D-A QS composite, and (c) the corresponding population-dependent TA dynamics at different photon energies.

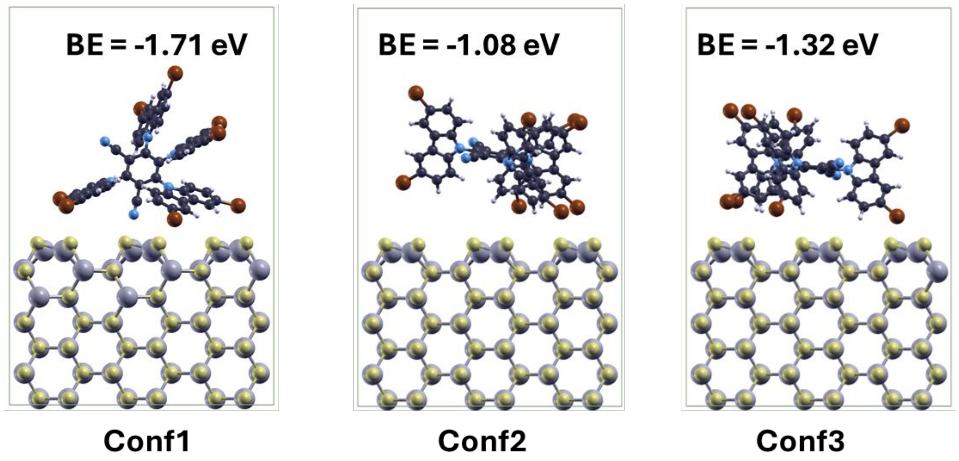

Figure S7. DFT-optimized geometries of the antenna molecule on the QS obtained from three different initial adsorption orientations. The binding energies of the optimized configurations are indicated.

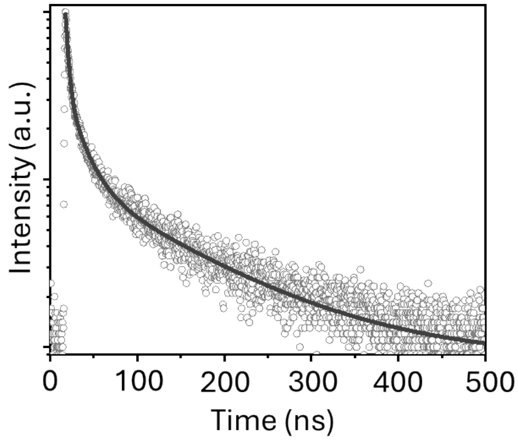

Figure S8. Radioluminescence decay profile of the D-A_4 composites at 665 nm.

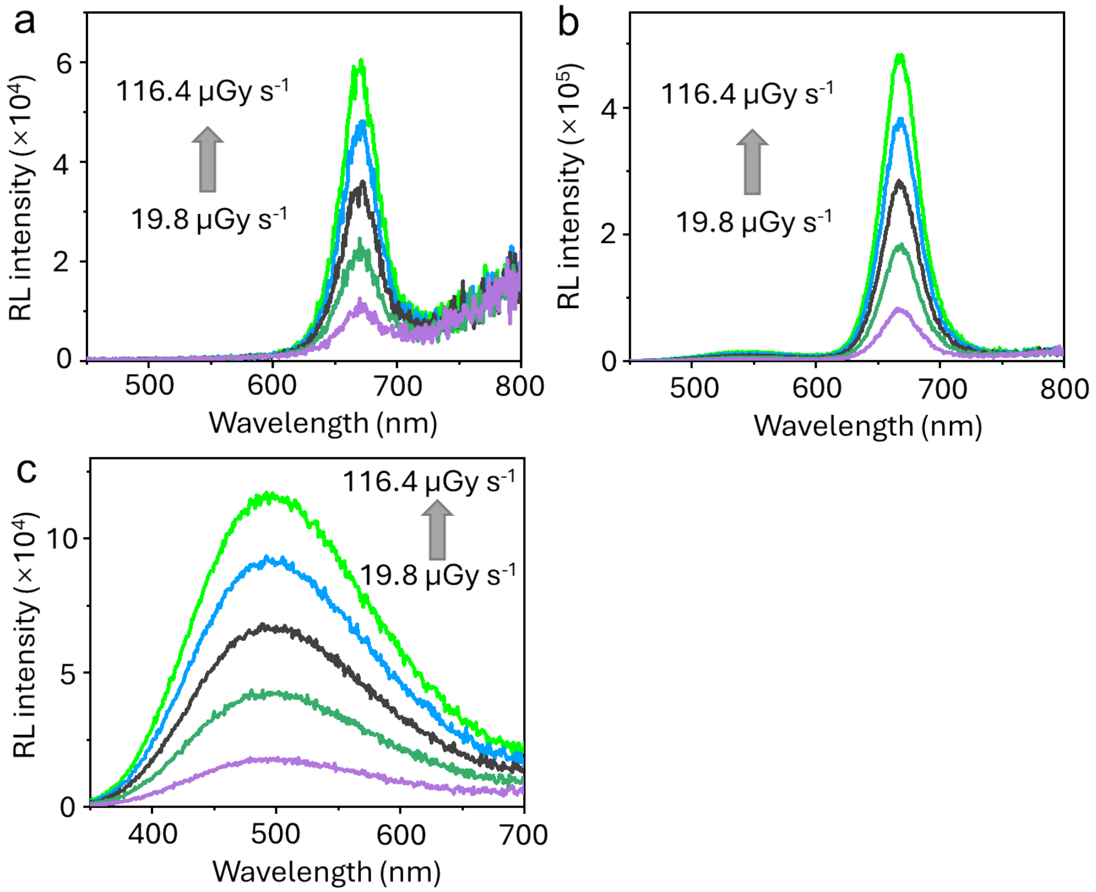

Figure S9. The dose rate-dependent radioluminescence spectra of (a) the A, (b) the D-A QS composite, and (c) the BGO.

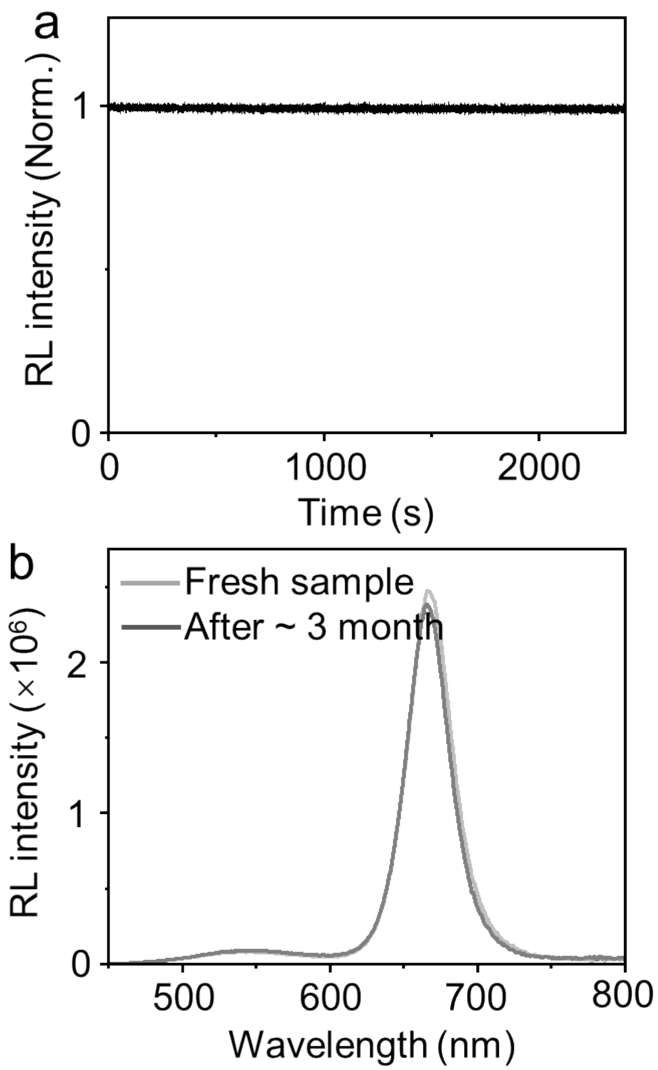

Figure S10. (a) RL spectra of the 4CzIPN-Br film under continuous X-ray irradiation at a dose rate of 0.25 mGy/s for 2400 seconds. (b) RL spectra of the D-A QS-composite film immediately after preparation and after 4 months of storage.

Table S1. Summary of the light yields of the composite systems and commercial scintillator.

Materials	Light yields (Photons/MeV)
QS-Composite	21000
QS	2200
BGO	10000

Table S2. Collected X-ray imaging resolution of the reported scintillators.

Materials	Imaging resolution (lp/mm)	Ref.
$Cs_3Cu_2I_5$/PDMS	17.0	10
TPE-4Br	18.7	11
Ln-BPTC	18.0	12
DMAc-TRZ:SO	16.6	8
Cu-doped Cs_2AgI_3/PDMS	16.2	13
PZ/PT	18.0	14
Tb-Cu_4I_4 MOFs	12.6	15
Tb-MOF-76	16.6	16
In-doped $Cs_3Cu_2I_5$ single crystals	18.0	17
BA_2PbBr_4: Mn/PMMA	10.7	18
DP/Br-D@PDMS	8.1	19
Y-PCN-94	14.3	20
TADF-Br – Ir-OMC composite	19.8	21
Zr-fcu-BADC-MOF – TADF composite	1.1	22
$CsPbBr_3$ NC/ceramic	15.0	23

References

1. Cassidy, J.; Ellison, C.; Bettinger, J.; Yang, M.; Moroz, P.; Zamkov, M. Enabling Narrow Emission Line Widths in Colloidal Nanocrystals through Coalescence Growth. *Chem. Mater.* **2020**, *32*, 7524-7534.

2. Kresse, G. F., J. Efficiency of Ab-Initio Total Energy Calculations for Metals and Semiconductors Using a Plane-Wave Basis Set. *Comput. Mater. Sci.* **1996**, *6*, 15-50.

3. Grimme, S.; Ehrlich, S.; Goerigk, L. Effect of the Damping Function in Dispersion Corrected Density Functional Theory. *J. Comput. Chem.* **2011**, *32*, 1456-1465.

4. Becke, A. D.; Johnson, E. R. A Density-Functional Model of the Dispersion Interaction. *J. Chem. Phys.* **2005**, *123*, 154101.

5. Xu, L. J.; Lin, X.; He, Q.; Worku, M.; Ma, B. Highly Efficient Eco-Friendly X-ray Scintillators Based on An Organic Manganese Halide. *Nat. Commun.* **2020**, *11*, 4329.

6. Zhou, Y.; Chen, J.; Bakr, O. M.; Mohammed, O. F. Metal Halide Perovskites for X-ray Imaging Scintillators and Detectors. *ACS. Energy. Lett.* **2021**, *6*, 739-768.

7. Chen, W.; Zhou, M.; Liu, Y.; Yu, X.; Pi, C.; Yang, Z.; Zhang, H.; Liu, Z.; Wang, T.; Qiu, J.; Yu, S. F.; Yang, Y.; Xu, X. All-Inorganic Perovskite Polymer–Ceramics for Flexible and Refreshable X-ray Imaging. *Adv. Funct. Mater.* **2021**, *32*, 2107424.

8. Ma, W.; Su, Y.; Zhang, Q.; Deng, C.; Pasquali, L.; Zhu, W.; Tian, Y.; Ran, P.; Chen, Z.; Yang, G.; Liang, G.; Liu, T.; Zhu, H.; Huang, P.; Zhong, H.; Wang, K.; Peng, S.; Xia, J.; Liu, H.; Liu, X.; Yang, Y. M. Thermally Activated Delayed Fluorescence (TADF) Organic Molecules for Efficient X-ray Scintillation and Imaging. *Nat. Mater.* **2022**, *21*, 210-216.

9. Wang, J.-X.; Gutiérrez-Arzaluz, L.; Wang, X.; He, T.; Zhang, Y.; Eddaoudi, M.; Bakr, O. M.; Mohammed, O. F. Heavy-Atom Engineering of Thermally Activated Delayed Fluorophores for High-Performance X-ray Imaging Scintillators. *Nat. Photon.* **2022**, *16*, 869-875.

10. Zhou, Y.; Wang, X.; He, T.; Yang, H.; Yang, C.; Shao, B.; Gutiérrez-Arzaluz, L.; Bakr, O. M.; Zhang, Y.; Mohammed, O. F. Large-Area Perovskite-Related Copper Halide Film for High-Resolution Flexible X-ray Imaging Scintillation Screens. *ACS Energy Lett.* **2022**, *7*, 844.

11. Du, X.; Zhao, S.; Wang, L.; Wu, H.; Ye, F.; Xue, K.-H.; Peng, S.; Xia, J.; Sang, Z.; Zhang, D.; Xiong, Z.; Zheng, Z.; Xu, L.; Niu, G.; Tang, J. Efficient and Ultrafast Organic Scintillators by Hot Exciton Manipulation. *Nat. Photon.* **2024**, *18*, 162-169.

12. Li, H.; Li, Y.; Zhang, L.; Hu, E.; Zhao, D.; Guo, H.; Qian, G. A Thermo-Responsive MOFs for X-Ray Scintillator. *Adv. Mater.* **2024**, *36*, e2405535.

13. He, T.; Zhou, Y.; Wang, X.; Yin, J.; Gutiérrez-Arzaluz, L.; Wang, J.-X.; Zhang, Y.; Bakr, O. M.; Mohammed, O. F. High-Performance Copper-Doped Perovskite-Related Silver Halide X-ray Imaging Scintillator. *ACS Energy Lett.* **2022**, *7*, 2753.

14. Zhou, Z.; Wang, X.; Lv, A.; Ding, M.; Song, Z.; Ma, H.; An, Z.; Huang, W. Achieving Efficient X-ray Scintillation of Purely Organic Phosphorescent Materials by Chromophore Confinement. *Adv. Mater.* **2024**, *36*, e2407916.

15. Liu, X.; Li, R.; Xu, X.; Jiang, Y.; Zhu, W.; Yao, Y.; Li, F.; Tao, X.; Liu, S.; Huang, W.; Zhao, Q. Lanthanide(III)-$Cu_{(4)}I_{(4)}$ Organic Framework Scintillators Sensitized by Cluster-Based Antenna for High-Resolution X-ray Imaging. *Adv. Mater.* **2023**, *35*, e2206741.

16. Zhang, X.; Qiu, H.; Luo, W.; Huang, K.; Chen, Y.; Zhang, J.; Wang, B.; Peng, D.; Wang, Y.; Zheng, K. High-Performance X-Ray Imaging using Lanthanide Metal-Organic Frameworks. *Adv. Sci.* **2023**, *10*, e2207004.

17. Wang, Q.; Zhou, Q.; Nikl, M.; Xiao, J.; Kucerkova, R.; Beitlerova, A.; Babin, V.; Prusa, P.; Linhart, V.; Wang, J.; Wen, X.; Niu, G.; Tang, J.; Ren, G.; Wu, Y. Highly Resolved X-Ray Imaging Enabled by In (I) Doped Perovskite-Like $Cs_3Cu_2I_5$ Single Crystal Scintillator. *Adv. Opt. Mater.* **2022**, 10, 2200304.

18. Shao, W.; Wang, X.; Zhang, Z.; Huang, J.; Han, Z.; Pi, S.; Xu, Q.; Zhang, X.; Xia, X.; Liang, H. Highly Efficient and Flexible Scintillation Screen Based on Manganese (II) Activated 2D Perovskite for Planar and Nonplanar High-Resolution X-Ray Imaging. *Adv. Opt. Mater.* **2022**, *10*, 2102282.

19. Xing, G.; Cui, E.; Yuan, X.; Wang, B.; Zhao, Y.; Tang, J.; Chen, J.; Liu, J. Defects in Ligand-Exchange-Passivated Mixed-Halide Double Perovskite Nanocrystals for X-ray Imaging. *Laser Photonics Rev.* **2024**, *18*, 2401552.

20. Zhang, L.; Wang, X.; Wang, X.; Wang, X.; Luo, Y.; Tan, C.; Jiang, L.; Wang, Y.; Liu, W. Fabrication of a Large-Area Flexible Scintillating Membrane for High-Resolution X-ray Imaging Using an AIEgen-Functionalized Metal-Organic Framework. *Inorg. Chem.* **2023**, *62*, 6421-6427.

21. Wang, J.-X.; Dutta, I.; Yin, J.; He, T.; Gutiérrez-Arzaluz, L.; Bakr, O. M.; Eddaoudi, M.; Huang, K.-W.; Mohammed, O. F. Triplet-triplet energy-transfer-based transparent X-ray imaging scintillators. *Matter.* **2022**, *6*, 217-225.

22. Wang, J.-X.; Gutiérrez-Arzaluz, L.; Wang, X.; Almalki, M.; Yin, J.; Czaban-Jóźwiak, J.; Shekhah, O.; Zhang, Y.; Bakr, O. M.; Eddaoudi, M.; Mohammed, O. F. Nearly 100% Energy Transfer at the Interface of Metal-Organic Frameworks for X-ray Imaging Scintillators. *Matter* **2022**, *5*, 253-265.

23. Ma, W.; Jiang, T.; Yang, Z.; Zhang, H.; Su, Y.; Chen, Z.; Chen, X.; Ma, Y.; Zhu, W.; Yu, X.; Zhu, H.; Qiu, J.; Liu, X.; Xu, X.; Yang, Y. M. Highly Resolved and Robust Dynamic X-ray Imaging Using Perovskite Glass-Ceramic Scintillator with Reduced Light Scattering. *Adv. Sci.* **2021**, *8*, 2003728.
